# Assessing the relationship between terrorist attacks against ingroup or outgroup members and public support for terrorism

**DOI:** 10.3389/fpsyg.2022.778714

**Published:** 2022-10-03

**Authors:** Sandy Schumann, Bettina Rottweiler, Paul Gill

**Affiliations:** Department of Security and Crime Science, University College London, London, United Kingdom

**Keywords:** public opinion, terrorism, war weariness, outgroup violence, time-series

## Abstract

Terrorist groups rely on constituency support for their long-term survival. Here, we examined the extent to which terrorists’ own activities are related with public opinion on terrorism. Specifically, we assessed whether more frequent and more costly terrorist attacks against the ingroup are associated with war weariness or retaliatory sentiments, thus, either weaker or stronger approval of terrorism. We further investigated if more frequent and costly attacks that target an outgroup predict higher levels of justification of terrorism. Lastly, we identified the timeframe during which domestic and outgroup terrorist attacks correlate with (lower or higher) public support. The analyses focused on Jordan (ingroup) and Israel (outgroup), over an 8-year period (2004–2011), drawing on data from the Pew Global Attitudes Survey and the Global Terrorism Database. Results showed that support for terrorism in Jordan decreased in 2005 and, again, in 2008. The frequency of terrorist attacks and fatality/injury rates in Jordan did not vary significantly during the study period. The number of attacks and fatalities/people injured in Israel, however, changed between 2004 and 2011. Cross-correlations of the time-series further demonstrated that the number of attacks and fatalities/people injured in Jordan was not related with the level of public approval of terrorism in the country. Importantly, and in line with the literature, the casualty rate in Israel was positively associated with support for terrorism in Jordan, in the next year. That is, there is evidence that more/less costly terrorist attacks on an outgroup can predict stronger/weaker public support for the tactic relatively quickly. Those findings provide insights for counter-terrorism measures.

## Introduction

“*The strongest weapon which the mujahedeen enjoy … is popular support from the Muslim masses in Iraq, and the surrounding Muslim countries. So we must maintain this support as best we can, and we should strive to increase it … In the absence of this popular support, the Islamic mujahed movement would be crushed in the shadows … The mujahed movement must avoid any action that the masses do not understand or approve*” (letter from Ayman al-Zawahiri to Abu Musab al-Zarqawi, who later led ISIS, released in October 2005; Global Security, 2005).

In the above quote, Al Qaeda’s former leader outlined that the success of the group’s plans at the time—defeating the U.S. Army in Iraq and establishing an Islamic Caliphate—depended on whether the public in Muslim-majority countries sympathizes with Al Qaeda. Cognizant that public opinion would be affected by Al Qaeda’s choice of tactics, al-Zawahiri insisted that the group should refrain from activities that could potentially diminish approval (see Sharvit et al., 2015). Although perhaps counter-intuitive, this position is unsurprising. To operate efficiently over a long period, terrorist groups rely on symbolic support (Mor, 1997; Paul, 2009; Schmid, 2017), that is, the public justifying acts of terrorism or endorsing terrorist groups and their actions. High, stable levels of public approval of terrorism in a territory can serve as an indicator of the perceived legitimacy of terrorist actors (Kruglanski and Fishman, 2006) and suggests the scale of the radical milieu (Malthaner and Waldmann, 2014) or complicit surround (Richardson, 2006) from which supporters can be drawn, enhancing the chance to establish and sustain (political) power (Bueno de Mesquita, 2005).

Several individual-level characteristics (e.g., religiosity, age, and gender) have been found to be associated with public support for terrorist activities (Fair and Shepherd, 2006; Tessler and Robbins, 2007). Less is known about the role of macro-level factors, namely, how terrorists’ own actions influence public opinion. Additionally, while it has been proposed that support for terrorism weaned since the early 2000s, systematic analyses of trends over time are rare (Pew Research, 2005; Wilke and Samaranayake, 2006; Lipka, 2017). A small number of longitudinal studies investigated the (oftentimes mobilizing) impact of attacks against outgroup members on public support for terrorism (e.g., Bloom, 2004; Jaeger et al., 2010, 2012; Sharvit et al., 2015). It remains, however, untested how domestic terrorist activities shape the approval of terrorism (Bueno de Mesquita and Dickson, 2007).

We aim to advance the current literature in three important ways. First, focusing on one Muslim-majority country—Jordan—we apply time-series analysis to investigate whether public approval of terrorism has indeed decreased between 2004 and 2011 (Pew Research, 2005; Wilke and Samaranayake, 2006; Lipka, 2017). Second, we test the association between terrorist activities and the observed trend in public opinion. Specifically, drawing on research that explored effects of exposure to violence (Berrebi and Klor, 2006; Jaeger et al., 2012; Canetti et al., 2017; Brouard et al., 2018; Aytaç and Çarkoğlu, 2021; Kupatadze and Zeitzoff, 2021; Godefroidt, 2022), we assess if more frequent and more costly (i.e., incurring more fatalities and injuries) domestic terrorist attacks predict the expected *decrease* in public support for terrorism in Jordan. Furthermore, we conceptually replicate previous work and determine the extent to which terrorist attacks that inflict more harm on an outgroup—here, Israel—are related with *stronger* approval of terrorism. Finally, we aim to clarify in which timeframe public opinion on terrorism is associated with the frequency and casualty rate of both domestic and outgroup attacks.

## Background

Previous research unanimously concluded that the majority of the public does not endorse terrorism (e.g., Tessler and Robbins, 2007; Pew Research Center, 2011; Poushter, 2015; Lipka, 2017; Schmid, 2017) and is highly concerned about extremism (Poushter, 2015). Differences in approval rates were documented when considering specific terrorist groups and their targets. For example, a 2014 poll showed that approximately one third of respondents in Kuwait, Saudi Arabia, and the United Arab Emirates reported very positive or fairly positive views of the Muslim Brotherhood. Support for ISIS ranged, in the same populations, between 3 and 5% (Pollock, 2014). Public support was also lower for terrorist attacks that targeted civilians compared to those targeting the U.S. military (Medoff and Ciolek, 2009; Shafiq and Sinno, 2010). Importantly, attacks against an outgroup were justified more strongly than attacks against ingroup members (Kaltenthaler et al., 2010).

The aforementioned findings rely on cross-sectional analyses and, therefore, represent snapshots of attitudes at a certain moment. As with all opinions, approval of terrorism is expected to change over time (see the Almond-Lippman consensus; Holsti, 1992). Notably, the Pew Global Attitudes Survey, which tracks indicators of endorsement of terrorism in several countries, indicated that (a) views of Hezbollah became increasingly unfavorable in five countries in the Middle East between 2007 and 2014, (b) approval of Hamas decreased among Palestinians in the same period, and (c) Osama bin Laden’s stock was reduced between 2003 and 2011 (Pew Research Center, 2011; Pew Research, 2014). Indeed, publications by the Pew Research Center have stipulated repeatedly that support for terrorism has decreased, especially in Muslim-majority countries, since the early 2000s (Wilke & Samaranayake, 2006; Pew Research Center, 2011; Lipka, 2017). However, these conclusions were, to our knowledge, not based on inferential statistical analyses. As such, it is unclear whether the observed differences in levels of support were not simply random variations, that is, neither statistically significant nor practically meaningful.

Documenting a decline in public support for terrorism is, of course, desirable. Equally important is understanding the factors that facilitate such a development. Previous research has highlighted the potential impact of terrorist activities that inflicts harm on an outgroup. More precisely, Jaeger et al. (2010) demonstrated that attacks that were committed by Hamas against Israeli targets predicted stronger public endorsement of the group among Palestinians (see also Bloom, 2004; Brym and Araj, 2008 failed to endorse this conclusion). Replicating this result, Sharvit et al. (2015) showed that, over the course of 6 years, a higher number of attacks targeting Israel was associated with stronger Palestinian public support for suicide bombings.

Modeling and simulation studies (Bueno de Mesquita and Dickson, 2007; Siqueira and Sandler, 2007) further suggest that support for terrorism can increase following domestic terrorist campaigns. Terrorist attacks may be used strategically to elicit government responses that the public—in particular those who terrorists seek to act for or who are already inclined to support terrorism—perceive to be out of proportion (*propaganda of the deed*; Bueno de Mesquita and Dickson, 2007). Aggrieved populations are then expected to endorse terrorist groups more strongly because counter-terrorism efforts affect them negatively. Specifying the potential implications of exposure to domestic terrorism, Hazlett (2020), drawing on Posen (1993), noted that experiences of violence convey that one’s community is victimized and that the enemy cannot be trusted. To defend oneself and the ingroup against those who could strike again, individuals justify further retaliatory action (see Hayes and McAllister, 2001; Canetti et al., 2017). Emotions—notably, the action-oriented emotion anger—were found to drive the association between exposure to violence and approval of further aggression (Lerner et al., 2003; Small et al., 2006; Hirsch-Hoefler et al., 2014; Fisk et al., 2019; Jost, 2019; Shandler et al., 2021). In line with this rationale, exposure to violence by foreign actors facilitated hardline foreign policy attitudes (Kupatadze and Zeitzoff, 2021) and negative opinions about an outgroup (Beber et al., 2014). Domestic terrorist attacks also fostered voting for right-wing political parties as well as agreement with more aggressive security policies (Berrebi and Klor, 2006; Bonanno and Jost, 2006; Hetherington and Suhay, 2011; Brouard et al., 2018; Jost, 2019; Aytaç and Çarkoğlu, 2021). Fielding and Penny (2009) further showed that support of the Oslo accord and the peace process decreased among Israelis following a rise in the number of attacks from Gaza and/or more Israeli fatalities. Support for the peace negotiations declined immediately after the attacks and remained low for 1 month. Moreover, Israeli violence that incurred Palestinian fatalities was associated with a reduction in support for moderate Palestinian political actors (i.e., Fatah) 1 month after the violence occurred. These effects were no longer identified after 2 months (Jaeger et al., 2010, 2012).

Thus far, it could be concluded that experiences of violence targeting either the outgroup or ingroup beget a stronger justification of violence. The *war weariness hypothesis* (Richardson, 1960), however, proposes an alternative account: those who are exposed to the destructive impact of conflict, high casualties or economic costs, are expected to endorse peaceful relations with conflict partners and be less sympathetic toward activities that prolong the violence (Gould and Klor, 2010; Blair et al., 2013; Zeitzoff, 2014). Underlying the rejection of violence should be feelings of threat (Huddy et al., 2003, 2005; Rubin et al., 2005). Specifically, while perceived collective threat likely increases calls for counter-aggressions, perceived personal threat predicts the recognition of compromises (Canetti et al., 2017). Indeed, support for militant groups in Pakistan was reduced when experiences of the costs of a conflict were more salient (Blair et al., 2013). Additionally, support for the insurgency that erupted after the U.S.-led invasion of Iraq in 2003 declined once attacks became deadlier and more frequent (Ciolek et al., 2006; Hafez, 2006).

## The present research

Taken together, there is evidence that public support for terrorism fluctuates over time, influenced by terrorist attacks that target an outgroup (e.g., Jaeger et al., 2010; Sharvit et al., 2015) as well as by domestic terrorist attacks (e.g., Bueno de Mesquita and Dickson, 2007; Hetherington and Suhay, 2011; Jost, 2019; Hazlett, 2020). The present study aims to build on and extend these insights. We focus on Jordan—the reference ingroup—and Israel, the outgroup. This choice of study context was guided by pragmatic and conceptual reasons. As will be described in more detail below, Jordan is one of two countries (the other being Turkey) for which data on public support for terrorism are available over an 8-year period. This relatively long time-series allows us to draw more robust conclusions about trends in public opinion. In addition, given Jordan’s historical experiences as well as demographic make-up, an unambiguous outgroup—Israel—could be identified. In 1994, the Israel-Jordan peace treaty was signed to end more than 4 decades of tense relationships and war between the countries. Jordan is also home to approximately 2 million Palestinian refugees. The Palestine Liberation Organization, indeed, led its activities in the 1960s from Jordan and was later driven out of the country. Hamas was also based in Jordan in the 1990s.

Applying time-series analysis, our research examined, first, if support for terrorism declined in Jordan between 2004 and 2011 (*Hypothesis 1*). Doing so, we provide first systematic empirical evidence of changes in public opinion on terrorism that extends beyond the mere inspection of raw data (see Wilke and Samaranayake, 2006; Pew Research Center, 2011; Lipka, 2017). Second, we investigate whether and how *domestic terrorist activity in Jordan* predicts the temporal fluctuation in public opinion. Specifically, we assess the extent to which a higher frequency and casualty rate of domestic terrorist attacks is associated with a reduction (*Hypothesis 2*) in public support for terrorism. Our analyses advance previous research that focused only on individual-level risk factors of the justification of terrorism (Fair and Shepherd, 2006; Tessler and Robbins, 2007). Considering a novel outcome variable, we also contribute to accounts that postulated either war weariness or retaliatory sentiments as a result of exposure to violence (Berrebi and Klor, 2006; Jaeger et al., 2012; Canetti et al., 2017; Brouard et al., 2018; Aytaç and Çarkoğlu, 2021; Kupatadze and Zeitzoff, 2021). Third, we determine if more frequent and more costly *attacks on an outgroup* (Israel) are associated with stronger endorsement of terrorism (*Hypothesis 3*). This analysis conceptually replicates a small number of studies conducted in the context of the Palestinian-Israeli conflict (Jaeger et al., 2010; Sharvit et al., 2015). Finally, we establish the timeframe in which domestic terrorist attacks or attacks that target an outgroup predict public approval of terrorism (*Research Question 1*). To our knowledge, only two studies have thus far explored this temporal pattern (Fielding and Penny, 2009; Jaeger et al., 2012), both pointing to immediate, short-term effects of terrorist activity on public opinion. We complement this work with evidence from a different study context to conclude whether terrorist attacks serve as a sustainable, or short-term, means to either attenuate or facilitate support for terrorism.

## Materials and methods

Our analyses were based on data from five time-series, described below. Each time-series was defined by eight time points with lags of 1 year (i.e., annual data from 2004 to 2011). Measures reflect the same operationalization of an indicator or same survey question at every wave.

To examine *public support for terrorism in Jordan*, we relied on the Pew Global Attitudes Survey (PGAS), a multi-country multi-wave public opinion survey. The PGAS was collected from 2002 to 2014. No data were available for 2003 and 2012. To allow for regular, 1-year lags in the time-series, data from 2002, 2013, and 2014 were excluded from the present research. The Pew Global Attitudes Survey is not a panel study, and new probability samples were drawn at each wave. However, samples in Jordan are representative of the adult population (i.e., representing 80% of the adult population). Thus, the public opinion data that defines the time-series is reliable at the aggregate level. At each wave, we excluded respondents who did *not* state that their religion was Islam (Table 1). This choice was informed by the phrasing of the public opinion measure, which made reference to the defense of Islam. Based on this exclusion criterium, on average *N* = 967 responses were considered in each wave.

**Table 1:** Overview of the number of responses per wave based on respondents’ religion.

**Religion**	**2004**	**2005**	**2006**	**2007**	**2008**	**2009**	**2010**	**2011**
**Muslim (included)**	964	967	972	965	968	963	968	971
**Christian (excluded)**	36	33	28	35	32	37	32	29

The PGAS captures public support for terrorism with the following item: “Some people think that suicide bombing and other forms of violence against civilian targets are justified in order to defend Islam from its enemies. Other people believe that, no matter what the reason, this kind of violence is never justified. Do you personally feel that this kind of violence is often justified to defend Islam, sometimes justified, rarely justified, or never justified?” Respondents could also indicate that they “do not know” or preferred not to answer the question. To prepare the data for further analysis, we first calculated in each wave the percentage of Muslim respondents who had endorsed each of the six answer options—“often,” “sometimes,” “rarely,” and “never justified” as well as “Do not know” and “refusal to answer” (Supplementary Material S1). Second, the sum of the percentages of respondents who reported that terrorism was either “often,” “sometimes,” or “rarely justified” was computed to reflect how many expressed that terrorism was “ever justified” (Table 2; see Fair and Shepherd, 2006). Doing so, respondents who refused to answer or stated that they did not know the answer were treated equal to those who stated that they considered terrorism as “never justified.” We adopted this approach as it ensured that the ratio of respondents who indicated that terrorism was “ever justified” was not artificially inflated.

**Table 2:** Public support for terrorism in Jordan per wave.

**Public opinion**	**2004**	**2005**	**2006**	**2007**	**2008**	**2009**	**2010**	**2011**
**Terrorism is “ever justified”**	94.8%	87.9%	56.4%	49.5%	53.9%	37.6%	45.2%	43.4%
**Terrorism is “never justified”**	2.5%	11.1%	43.1%	42.4%	40.9%	56%	53.8%	54.6%

Values are rounded.

To explore the implications of *domestic and outgroup terrorist attacks*, we took into account the overall number as well as the casualty rate (i.e., number of people killed or injured) of attacks. Indeed, it is perhaps not attacks *per se* but rather their costs that shape sentiments of perceived threat or anger (Getmansky and Zeitzoff, 2014; Huff and Kertzer, 2018) and, thus, predict public opinion. Additionally, it may be argued that attacks are more salient if they are more costly. By separating data on attack frequency and costs, we were able to acknowledge these dynamics. We identified the *number of terrorist attacks* (Table 3)*, fatalities and injuries* (Table 4) in Jordan and Israel by relying on the Global Terrorism Database (2020) (GTD; all data were created using the same event classification method). We only considered incidents that aimed at attaining a political, economic, religious, or social goal. Ambiguous and unsuccessful attacks were extracted as well. We further examined the RAND Database of Worldwide Terrorism Incidents (2021) (RDWTI) to verify information from the GTD till 2010, after which no data are available in the RDWTI. For Jordan, the number of attacks and casualties reported in both databases largely aligned. For Israel, however, data varied, with the RDWTI presenting a substantially higher number of attacks and fatalities. Additionally, we explored a recently released dataset that focuses specifically on jihadist attacks in Jordan (Gråtrud, 2021); here, more attacks were recorded than in the GTD. However, no data that relied on the same coding protocol are available for Israel. In order to conduct the analyses for both countries with comparable data, we used the GTD data. We acknowledge that these numbers may represent a conservative estimate. The hypotheses proposed a lagged relationship between domestic or outgroup terror attacks and public opinion. To reflect this rationale, we took into account the period in which the PGAS was administered in each year and then considered the attacks that had occurred between that and the previous data collection phase (see Supplementary Material S2 for details). For instance, in 2005, the PGAS was run between May 3 and May 24; in 2004, data were collected between February 24 and 29. Attack data that were matched with opinion data from 2005, therefore, included actions that took place between March 1, 2004 and May 2, 2005.

**Table 3:** Number of attacks in Jordan and Israel per wave.

**Country**	**2004**	**2005**	**2006**	**2007**	**2008**	**2009**	**2010**	**2011**
**Jordan**	0	0	3	1	0	0	1	1
**Israel**	37^*^	25	44	55	76	129	8	16

This data were based on incidents that occurred between February 23, 2003 and February 22, 2004. No PGAS was collected in 2003. The cut-off data of the 23rd February was chosen based on when data collection took place in 2004.

**Table 4:** Number of people injured/killed (combined score) in Jordan and Israel per wave.

**Country**	**2004**	**2005**	**2006**	**2007**	**2008**	**2009**	**2010**	**2011**
**Jordan**	0	0	163	7	0	0	0	5
**Israel**	726^*^	227	236	185	61	261	19	14

This data were based on incidents that occurred between February 23, 2003 and February 22, 2004. No PGAS was collected in 2003. The cut-off data of the 23rd February were chosen based on when data collection took place in 2004.

## Results

The code to reproduce the analyses as well as the raw data of public opinion are presented in the Supplementary Materials S1, S3. All analyses were conducted with *R* 3.6.3, and relevant packages are specified in the analysis scripts.

### Analytical approach

The analytical approach encompassed three steps. First, we examined whether and how public support for terrorism in Jordan changed during the study period. Second, we assessed the temporal variation of the frequency of terror attacks as well as the resulting number of fatalities/injuries, both in Jordan and Israel. Third, we tested the extent to which the five time-series (public opinion and frequency of terror attacks in Jordan, public opinion and frequency of terror attacks in Israel, public opinion and casualties in Jordan, public opinion and casualties in Israel) were correlated.

More precisely, in step one and two, we sought to identify the structure underlying the time-series data. Our hypotheses presumed a variation in public opinion predicted by variation in the number of terror attacks or fatalities/people injured. To detect the nature of these temporal variations, we compared three alternative data structures and determined which one offered the best fit (or smallest discrepancy) from the observed data (Kleinberg et al., 2020). We fitted an intercept-only model, a linear temporal trend model, and a structural breakpoint model for each time-series. The intercept-only model assumed that, for instance, public support for terrorism did not change during the study period (i.e., the regression coefficient in the model was zero). The linear trend model stipulated a strictly linear progression with a stable non-zero regression coefficient. The structural breakpoint model proposed *n* structural breaks at which the non-zero regression coefficient changed significantly, that is, significant points of change in the trend of opinion, attack frequency, or casualty rate. The number and position of the breakpoints in the time-series was not pre-determined. To extract this information, Bayesian Information Criterion (BIC) plots were examined and the breakpoint model (as well as the other two models) were plotted against the observed data (Zeileis et al., 2003). The three models are non-nested. They were therefore compared using the Akaike’s Information Criterion (AIC; Akaike, 1974), BIC, as well as the mean absolute error (MAE), and the root mean squared error (RMSE). Better model fit, that is, less discrepancy from the observed data, was indicated by lower AIC, BIC, MAE, and RMSE values.

To explore whether, and in which way, the number of terrorist attacks and casualty rates in Jordan and Israel predicted public support for terrorism in Jordan, we first established whether the time-series were stationary. A stationary time-series suggests that the properties that generate the structure of the time-series remain stable over time such that the distribution of the data does not change when time passes. The Kwiatkowski-Phillips-Schmidt-Shin (KPSS) test was conducted. The KPSS test examines the null hypothesis that the time-series is level stationary. Finally, we calculated the cross-correlation between the respective stationary time-series. The cross-correlation function also specifies the lag of the relationships, that is, whether detected associations are present at the same or across specific waves. When interpreting the lag, we considered that data was structured such that attack frequency/fatality rates stated in the same wave as PGAS data referred in fact to events that occurred (approximately) over the previous year. To further determine the direction of the relationship indicated through the cross-correlation, the Granger causality (Granger, 1969) was then examined. Granger causality tests determine whether past values of one time-series allow, or rather improve, the forecasting of another time-series, beyond past information of the dependent series (Barnett and Seth, 2014).

### Assessing changes in public support for terrorism over time

**Figure 1:**
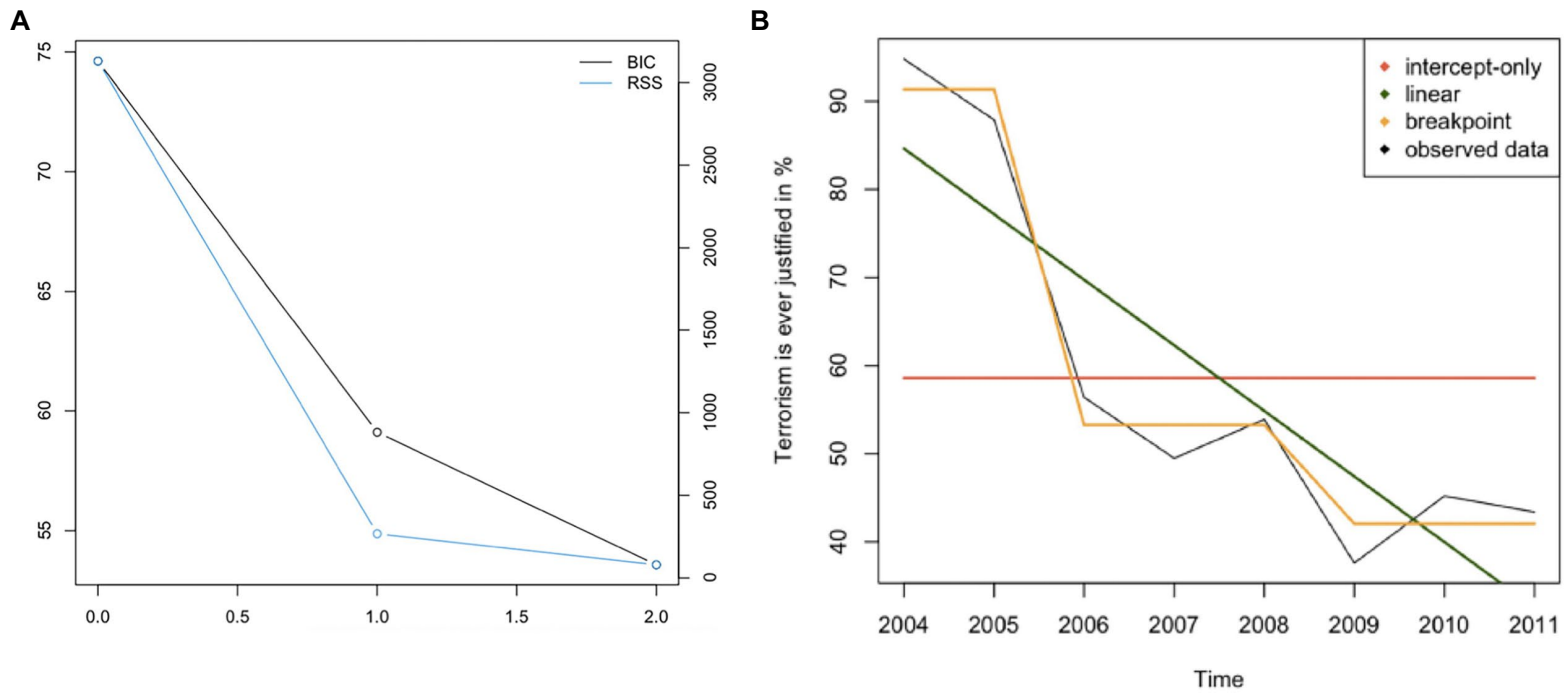
Panel **(A)** – BIC plot, Panel **(B)** - Plotting observed data and models for the time-series “Terrorism is ever justified.”

Table 5 highlights that a breakpoint model fitted the data of public support for terrorism best. The MAE of 3.05 suggests that the average discrepancy between the observed data and values predicted by the breakpoint model is 3.05%. The BIC plot further demonstrated two structural breakpoints (Figure 1B). Plotting the intercept-only, linear and breakpoint models against the observed data revealed that the structural breaks occurred in 2005 and 2008 (Figure 1A). The percentage of people who reported that suicide terrorism was “ever justified” dropped between 2005 and 2006 from 87.9 to 56.4% and again in 2008 from 53.9 to 37.6% (in 2009). From 2009 onwards, the level of justification of terrorism remained at a lower level (see Table 2). Hypothesis 1 was not rejected.

**Table 5:** Model fit for all time-series.

**Time-series**	**Model**	**AIC**	**BIC**	**MAE**	**RMSE**
**Terrorism is “ever justified”**	Intercept-only	74.46	74.62	16.38	19.78
	Linear	65.57	65.81	9.24	10.02
	**Breakpoint (2)**	**49.10**	**49.42**	**2.92**	**3.16**
**Number of terrorist attacks Jordan**	**Intercept-only**	**26.17**	**26.35**	**0.75**	**0.97**
	Linear	28.16	28.40	0.73	0.97
	Breakpoint (0)	26.40	26.64	0.5	0.87
**Number of fatalities/people injured Jordan**	**Intercept-only**	**90.35**	**90.51**	**35.28**	**53.40**
	Linear	91.89	92.13	33.81	51.90
	Breakpoint (0)	90.34	90.58	28.97	47.11
**Number of terrorist attacks Israel**	Intercept-only	84.27	84.42	28.28	36.52
	Linear	86.26	86.5	28.08	36.50
	**Breakpoint (2)**	**74.39**	**74.71**	**12.13**	**15.34**
**Number of fatalities/people injured Israel**	Intercept-only	112.58	112.74	146.38	214.26
	**Linear**	**107.29**	**107.53**	**115.27**	**135.88**
	Breakpoint (1)	109.16	109.39	135.88	152.67

The model that provides the best fit is highlighted in bold.

### Assessing changes in the frequency and casualty rate of terrorist attacks

**Figure 2:**
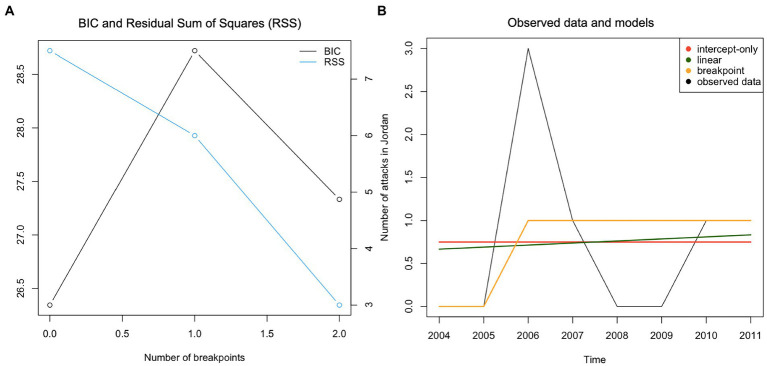
Panel **(A)** – BIC plot, Panel **(B)** - Plotting observed data and models for the time-series “Number of attacks in Jordan.”

**Figure 3:**
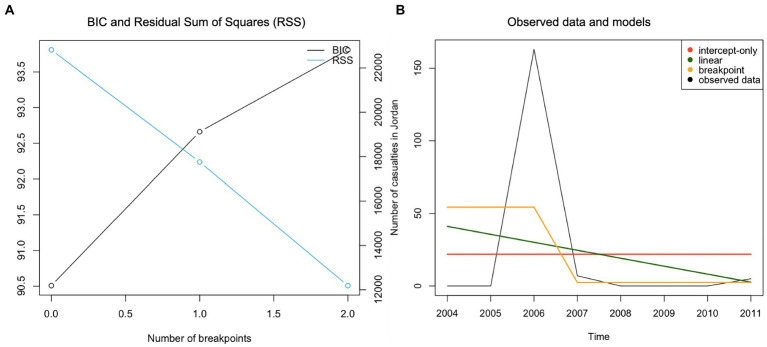
Panel **(A)** – BIC plot, Panel **(B)** - Plotting observed data and models for the time-series “Number of casualties in Jordan.”

Considering the *number of terrorist attacks in Jordan*, model fit indices indicated that the intercept-only model provided the best fit (Table 5; Figure 2); there were no significant changes in the number of attacks over the 8 years. Next, we applied the same procedure to the time-series of *number of fatalities/injuries in terrorist attacks in Jordan*. Upon initial inspection, the breakpoint model achieved the best fit. However, the BIC plot highlighted that no breakpoints were identified (Figure 3A). Therefore, it was concluded that the more parsimonious intercept-only model represented the observed data best (Table 5; Figure 3B).

**Figure 4:**
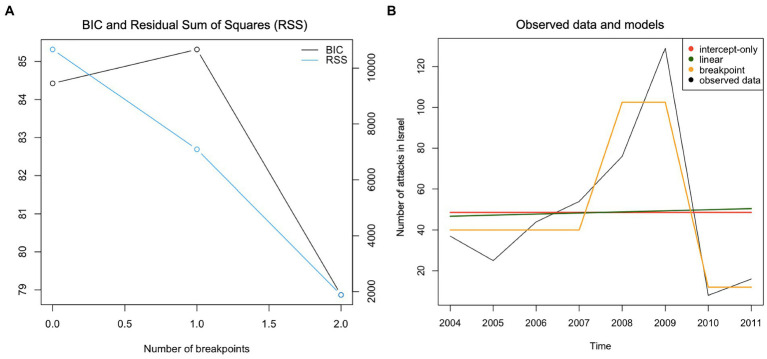
Panel **(A)** – BIC plot, Panel **(B)** - Plotting observed data and models for the time-series “Number of attacks in Israel.”

**Figure 5:**
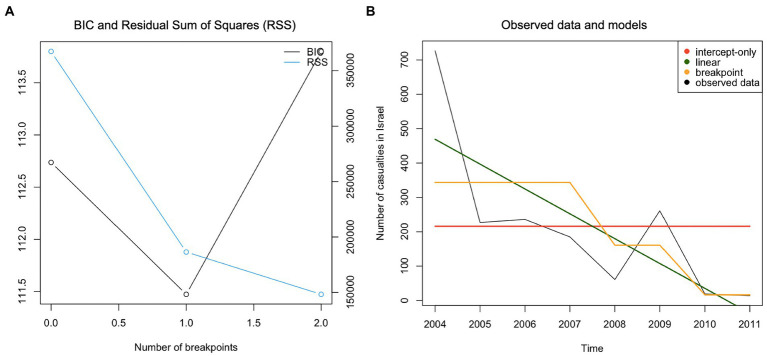
Panel **(A)** - BIC plot, Panel **(B)** - Plotting observed data and models for the time-series “Number of casualties in Israel.”

For *Israel*, the breakpoint model with two breaks offered the best fit to describe the development of *number of terrorist attacks* over time (Figure 4). Significantly more events were recorded between 2007 and 2008 than in the previous year; there were fewer incidents between 2009 and 2010 than between 2008 and 2009. Assessing the *casualty rates in Israel*, a linear model with a negative slope described the observed data best (Figure 5).

### Cross-correlations of time-series

The aforementioned analyses identified temporal variation in public support for terrorism in Jordan. In other words, it was justified to calculate cross-correlations to further explore predictors of the observed reduction in approval. The KPSS tests suggested that time-series were stationary (public opinion: KPSS level = 0.37, *p* = 0.09; number of attacks Jordan: KPSS level = 0.14, *p* > 0.10; fatalities and people injured Jordan: KPSS level = 0.19, *p* > 0.10; number of attacks Israel: KPSS level = 0.14, *p* > 0.10; and fatalities and people injured Israel: KPSS level = 0.41, *p* = 0.07). The time-series were therefore not differenced.

**Figure 6:**
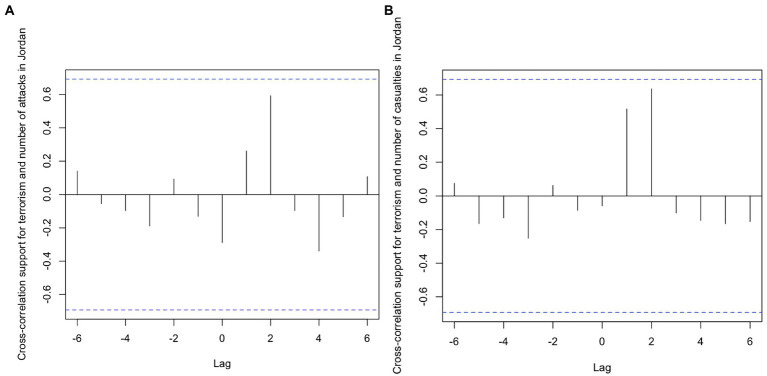
Cross-correlation function of time-series “Terrorism is ever justified” and “Number of attacks” Panel **(A)** as well as “Casualties in Jordan” Panel **(B)**.

**Figure 7:**
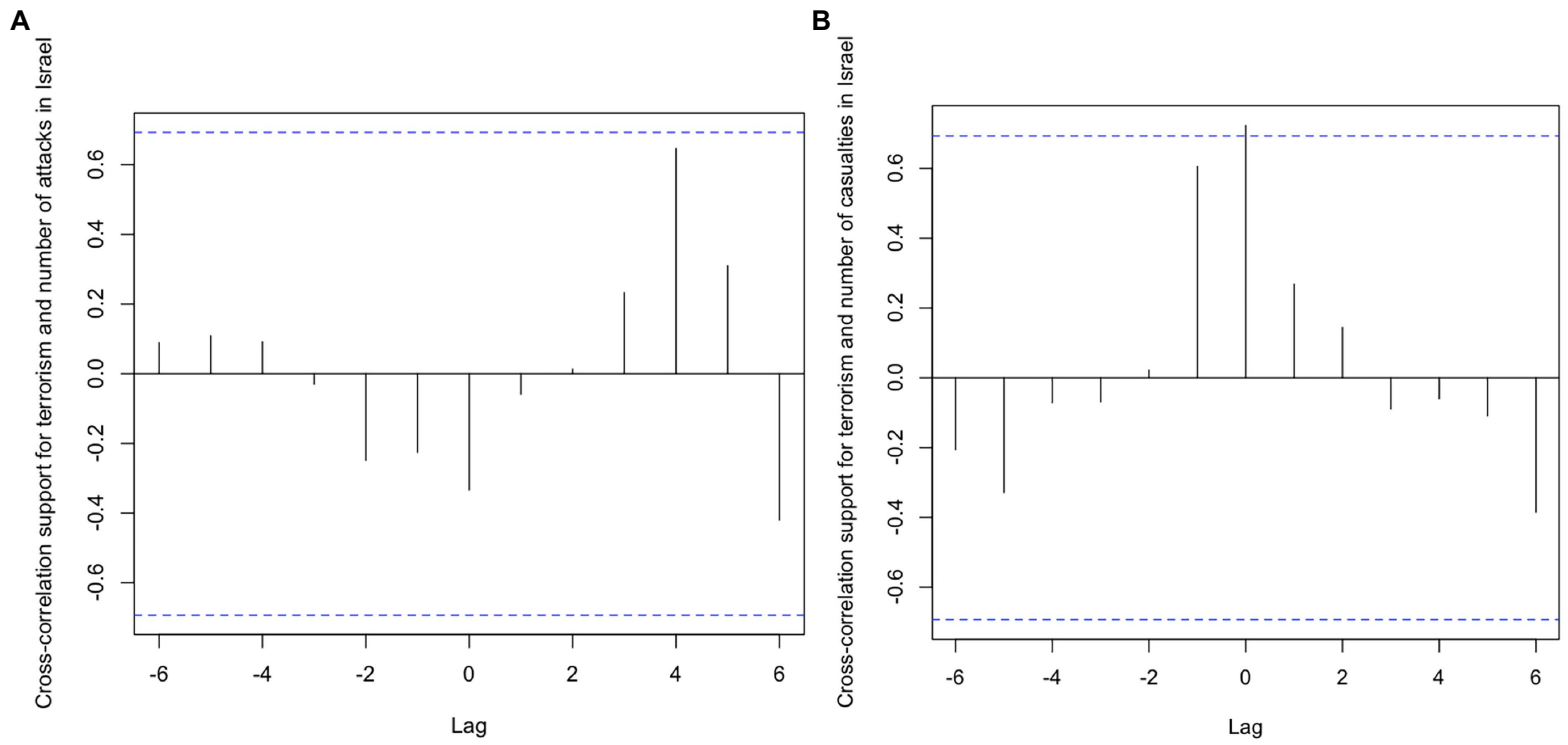
Cross-correlation function of time-series “Terrorism is ever justified” and “Number of attacks” Panel **(A)** as well as “Casualties in Israel” Panel **(B)**.

Analysis of the cross-correlation functions (Figure 6) showed no significant relationship between public support for terrorism and the number of attacks and casualty rates in Jordan. This result is not surprising, given that no significant changes were observed in the two predictor time-series. Moreover, the number of attacks in Israel was not related with approval of terrorism (Figure 7A). However, public opinion and the number of fatalities/injuries in Israel was strongly positively correlated (*r* = 0.72; *R*^2^ = 0.52 Figure 7B) at lag zero. Answering *Research Question* 1, the result pointed to a simultaneous association between approval of terrorism in Jordan and the number of casualties in terrorist attacks in Israel. It must be noted again that the attack data referred to a period spanning approximately 1 year before opinion data was collected; it does not represent attacks that occurred in the same year. The test for granger causality then indicated that a higher fatality/injury rate in Israel predicted stronger support for terrorism in Jordan (*F*(−1) = 16.73, *p = 0*.015). The reverse relationship—public opinion predicting fatality/injury rates in Israel—was not supported [*F*(−1) = 4.41, *p =* 0.104]. Hypotheses 2a and 2b were both rejected; Hypothesis 3 was not rejected.

## Discussion

Taken together, we showed that public support for terrorism in Jordan decreased significantly over an 8-year period in the early 2000s. Applying time-series analyses, we confirmed previous descriptive results (Wilke and Samaranayake, 2006; Pew Research Center, 2011; Lipka, 2017) to conclude that approval of terrorism is dynamic and subject to change over time. The fact that a breakpoint model best fitted the observed data provided initial evidence that unique critical events or accumulative trends affected the public’s opinion.

Notably, conceptually replicating Jaeger et al. (2010) and Sharvit et al. (2015) results, we found a positive relationship between casualty rates of outgroup attacks and the justification of terrorism; outgroup attack frequency was not correlated with public opinion. The differential findings for the predictors ‘outgroup attack frequency’ and ‘outgroup attack casualty rate’ could suggest that the level of the endorsement of terrorism varies in response to the mere salience of outgroup violence, which is expected to be higher for more costly actions. The one-year lagged relationship between outgroup casualty rate and public opinion also indicates that only more recent—or, again, perhaps more salient—costly outgroup attacks predict approval of terrorism. More precisely, it seems conceivable that more costly outgroup attacks serve as a reminder of a conflict with a specific outgroup, or the outgroup itself, that elicits a short-lived sentiment that terrorism is an acceptable or necessary tactic (to address the salient intergroup conflict). Further research is needed to investigate the role of outgroup and conflict salience in more detail.

Analyses of Grangar causality did not confirm the reverse direction of the relationship between outgroup terror attacks and endorsement of terrorism. This result contests work that suggested that terrorist activity itself is impacted by public opinion. Sharvit et al. (2015), for example, showed that higher levels of approval of violence in Palestine predicted a larger number of future attacks on Israel. Bloom (2004) also recognized that in the period after November 2000, different actors used suicide attacks on Israeli targets to compete over Palestinians’ support. One caveat of our study is that we did not extract whether the actors that committed attacks in Israel did indeed see the Jordanian public as a key stakeholder. We encourage further analyses of the respective attacks to conduct a more nuanced assessment.

Contrary to our hypothesis, we failed to identify a cross-correlation between the frequency of attacks and casualty rates in Jordan and levels of endorsement of terrorism. That is, although it has been noted that exposure to violence could evoke a need for retaliation or war weariness (Berrebi and Klor, 2006; Jaeger et al., 2012; Canetti et al., 2017; Brouard et al., 2018; Aytaç and Çarkoğlu, 2021; Kupatadze and Zeitzoff, 2021), which would suggest either a positive or negative association between domestic attacks and support for terrorism, we found no significant relationships. One way to interpret the finding is to consider the potential of cognitive and emotional desensitization. After being confronted with attacks over a longer period individuals may come to believe that terrorism is normal (i.e., cognitive desensitization). Attacks then do not elicit an emotional response such as fear or anger (i.e., emotional desensitization), and public opinion on terrorism may not fluctuate (Funk et al., 2004; see Castanho, 2018; Nussio, 2020). While appealing, this rationale does not appear suitable for the present context. Jordan has experienced overall low levels of domestic terrorism in the study period. Failure to detect a significant cross-correlation with this predictor is, therefore, likely due to the low level of variation of the time-series. Moreover, when examining the targets of attacks in Jordan, it is evident that three of seven known targets include foreign military and diplomatic staff; those attacks might, in fact, not have been viewed as ingroup attacks (Supplementary Material S5). In contrast, the large majority of attacks in Israel targeted Israeli citizens, military, infrastructure etc. (Supplementary Material S7), thus, are clearly categorized as outgroup attacks. We recommend that future research replicates our analysis in a context with a higher variability of domestic terrorist events and a higher percentage of ingroup attacks.

## Limitations

The aforementioned conclusions must be considered in light of the following limitations. First, due to not weighing the raw PGAS data to adjust for the probability of being included in the study or the sample design, we must acknowledge that the samples do not fully represent the population from which they were drawn. Specifically, respondents who describe their national group as Palestinian were oversampled in the PGAS, and it could be speculated that the trends in public opinion in our sample are more strongly influenced by this group than is evident in the Jordanian population as a whole. However, analyses presented in the Supplementary Material S4 show no systematic differences in support for terrorism between Palestinian and Jordanian respondents. Moreover, the measure examining public support for terrorism does not refer to active support provided to terrorist actors. As such, one should not draw conclusions about the degree of radicalization. To approximate the latter, dedicated questions regarding respondents’ own willingness to use violence to attain political, religious, or social justice goals must be included in public opinion polls. For ethical and legal reasons this is, understandably, not always feasible. Relatedly, the PGAS data were collected through interviews. As can be seen in the raw data (Supplementary Material S1), very few people refused to answer the question. However, it could be expected that concerns of social desirability affected the answers that were given, such that overall levels of approval of terrorism might be underestimated.

Additionally, it is worthwhile to reflect on the validity of the measure of public support for terrorism. The question that was used to examine public opinion did not make explicit reference to ingroup or outgroup members as victims. We only included respondents who described their religion as Muslim. As the question noted the need to use violence to defend Islam, respondents might have considered it to mean violence toward those who are not Muslim rather than the justification of terrorist tactics. Unfortunately, the present research does not allow us to clarify this matter further. Subsequent studies could, however, address this gap in the literature by including measures on ingroup, outgroup, and perpetrator perceptions as well as support for terrorism and investigate their discriminant validity.

Alternative, or complementary predictors of public support for terrorism at the individual, meso or macro level were not considered in our analysis. For example, the costs of attacks were only conceptualized as casualty rates. Economic costs, which may outweigh non-economic costs, were not introduced (Grossman et al., 2018; Manekin et al., 2019). Changes in attack tactics are a further potential confounding variable. That is, certain weapons or attack methods against outgroups may be justified less strongly. However, as shown in the Supplementary Material S8, no systematic variation in weapon type was identified for attacks in Israel during the study period. Time-invariant factors of the attacks were also not held constant, such as the group who committed the attacks, or broader social and political trends, namely, recessionary economic trends that have found to predict support for terrorism (Bueno de Mesquita, 2005; Bueno de Mesquita and Dickson, 2007).

Moreover, manifestation of other forms of violence, including violence from state actors or organized crime groups, in Jordan were not assessed; neither did we examine the impact of terrorist attacks against other outgroups and countries. These experiences could have also elicited a sentiment of war weariness that might be generalized to predict (better than terrorist attacks) the reduction of public support for terrorism. In light of the reduced complexity of our models, the presented associations therefore may be overestimated. The latter might also be the case because we chose one specific outgroup whose relationship with Jordan has been defined by a long-standing conflict. It is possible that when examining attacks on other outgroups, for example, non-Muslim majority countries with whom no direct conflict has been experienced, the strength of the association with public opinion could be weaker. Finally, as we identified discrepancies in the number of reported terrorist attacks in Israel between two datasets, we must acknowledge that this data could differ from the attacks that occurred and, further, from attacks that were recognized by the public in Jordan. Survey studies that examine what attacks people recalled could be a tool to overcome this concern.

Despite these limitations, we believe that the study makes relevant contributions to the literature. We highlighted the importance of assessing public approval of terrorism as a dynamic concept that changes over time. In addition, we showed that more costly terrorist attacks that target an outgroup can affect, fairly quickly, how strongly terrorist tactics are endorsed. Both terrorist and state actors are keen to direct public opinion in their favor (Schuurman, 2013). In light of our results, strategies that influence the public need to consider not only domestic events but, especially, activities that target outgroups.

## Data availability statement

The raw data supporting the conclusions of this article are available in the Supplementary material.

## Author contributions

SS conceptualized the study, conducted the literature review and analyses, and prepared a first draft of the article. BR extracted the data and cleaned datasets as well as revised the manuscript. PG revised the manuscript. Funding for the study was awarded to PG. All authors contributed to the article and approved the submitted version.

## Funding

Funding for the open access publishing as well as to carry out the research was provided by the European Research Council under the European Union’s Horizon 2020 Research and innovation program (Grant 75883), awarded to PG.

## Conflict of interest

The authors declare that the research was conducted in the absence of any commercial or financial relationships that could be construed as a potential conflict of interest.

## Publisher’s note

All claims expressed in this article are solely those of the authors and do not necessarily represent those of their affiliated organizations, or those of the publisher, the editors and the reviewers. Any product that may be evaluated in this article, or claim that may be made by its manufacturer, is not guaranteed or endorsed by the publisher.
