# Supplementary material for: Assessing the relationship between terrorist attacks against ingroup or outgroup members and public support for terrorism
Source: Front Psychol. 2022 Oct 3;13:778714. doi: 10.3389/fpsyg.2022.778714 (PMC9575669; doi:10.3389/fpsyg.2022.778714)
Supplement: Supplementary file 1 [file Table_1.DOCX]

**Supplementary Material**

**S1. Public Opinion Data in %**

| **Answer** | **2004** | **2005** | **2006** | **2007** | **2008** | **2009** | **2010** | **2011** |
| --- | --- | --- | --- | --- | --- | --- | --- | --- |
| **Often justified** | 32.7 | 24.3 | 4.6 | 5.8 | 7 | 3.7 | 8.5 | 3.5 |
| **Sometimes justified** | 50.1 | 32.6 | 24.1 | 16.7 | 17.8 | 7.7 | 12.1 | 9.1 |
| **Rarely justified** | 12 | 31 | 27.7 | 27 | 29.1 | 26.2 | 24.6 | 30.8 |
| **Never justified** | 2.5 | 11.1 | 43.1 | 42.4 | 40.9 | 56 | 53.8 | 54.6 |
| **Don't know** | 1.3 | 0.8 | 0.2 | 3.5 | 5 | 6.4 | 0.8 | 1.9 |
| **Refused** | 0.4 | 0.2 | 0.3 | 4.6 | 0.2 |  | 0.2 | 0.2 |

**S2. Timeline of data collection points and attacks**

| **Year** | **Public opinion data collection** | **Attacks Jordan** | **Attacks Israel** |
| --- | --- | --- | --- |
| 2004 |  |  | Jan-01 |
|  |  |  | Jan-14 |
|  |  |  | Jan-19 |
|  |  |  | Jan-29 |
|  |  |  | Feb-22 |
|  | February 24-29 |  |  |
|  |  |  | Mar-04 |
|  |  |  | Mar-06 |
|  |  |  | Mar-14 |
|  |  |  | Apr-17 |
|  |  |  | Jun-08 |
|  |  |  | Jun-20 |
|  |  |  | Jun-28 |
|  |  |  | Jul-11 |
|  |  |  | Jul-31 |
|  |  |  | Aug-01 |
|  |  |  | Aug-26 |
|  |  |  | Sep-29 |
|  |  |  | Nov-01 |
| 2005 |  |  | Jan-02 (2 counts) |
|  |  |  | Jan-07 |
|  |  |  | Jan-12 |
|  |  |  | Jan-14 |
|  |  |  | Jan-25 |
|  |  |  | Mar-20 |
|  |  |  | Apr-05 |
|  |  |  | Apr-07 |
|  |  |  | Apr-10 |
|  |  |  | Apr-12 |
|  |  |  | Apr-15 |
|  | May 3-24 |  |  |
|  |  |  | May-11 |
|  |  |  | Jun-07 |
|  |  |  | Jul-12 |
|  |  |  | Jul-23 |
|  |  |  | Aug-04 |
|  |  |  | Aug-08 |
|  |  | Aug-19 | Aug-24 |
|  |  |  | Aug-25 |
|  |  |  | Aug-28 |
|  |  |  | Oct-26 |
|  |  |  | Oct-28 |
|  |  | November 9 (3 counts) | Dec-05 |
|  |  |  | Dec-12 |
|  |  |  | Dec-18 |
|  |  |  | Dec-22 |
| 2006 |  |  | Jan (date unclear) |
|  |  |  | Jan-19 |
|  |  |  | Jan-31 |
|  |  |  | Feb-2 (2 counts) |
|  |  |  | Feb-3 (2 counts) |
|  |  |  | Feb-05 |
|  |  |  | Feb-07 |
|  |  |  | Feb-08 |
|  |  |  | Feb-10 (2 counts) |
|  |  |  | Feb-12 |
|  |  |  | Feb-14 (2 counts) |
|  |  |  | Feb-15 |
|  |  |  | Feb-20 |
|  |  |  | Feb-22 |
|  |  |  | Feb-23 |
|  |  |  | Feb-24 (3 counts) |
|  |  |  | Feb-25 |
|  |  |  | Mar-28 |
|  |  |  | Apr-1 (2 counts) |
|  |  |  | Apr-02 |
|  |  |  | Apr-4 (2 counts) |
|  | April 5 - 27 |  | Apr-06 |
|  |  |  | Apr-7 (3 counts) |
|  |  |  | Apr-09 |
|  |  |  | Apr-10 |
|  |  |  | Apr-17 |
|  |  |  | May-18 (2 counts) |
|  |  |  | May-25 |
|  |  |  | May-26 |
|  |  |  | May-28 |
|  |  |  | May-31 |
|  |  |  | Jun-01 |
|  |  |  | Jun-25 |
|  |  |  | Jul-04 |
|  |  |  | Jul-5 (2 counts) |
|  |  |  | Jul-12 (4 counts) |
|  |  |  | Jul-14 |
|  |  |  | Jul-24 |
|  |  |  | Jul-27 |
|  |  |  | Jul-28 |
|  |  |  | Aug-2 (2 counts) |
|  |  |  | Aug-3 (2 counts) |
|  |  | Sep-04 | Aug-06 |
|  |  |  | Sep-17 |
|  |  |  | Sep-18 |
|  |  |  | Sep-20 (2 counts) |
|  |  |  | Sep-21 (2 counts) |
|  |  |  | Sep-26 |
|  |  |  | Sep-27 (2 counts) |
|  |  |  | Sep-30 |
|  |  |  | Oct-06 |
|  |  |  | Nov-07 |
|  |  |  | Nov-12 |
|  |  |  | Nov-15 |
|  |  |  | Nov-19 |
|  |  |  | Nov-26 |
|  |  |  | Nov-27 |
|  |  |  | Nov-28 |
|  |  |  | Dec-27 |
| 2007 |  |  | Jan-28 |
|  |  |  | Jan-29 |
|  |  |  | Feb-21 |
|  |  |  | Feb-24 |
|  |  |  | Mar-17 |
|  | April 9 - May 7 |  | Apr-10 |
|  |  |  | Apr-14 |
|  |  |  | Apr-24 |
|  |  |  | May-06 |
|  |  |  | Jun-1 (2 counts) |
|  |  |  | Jun-2 (2 counts) |
|  |  |  | Jun-04 |
|  |  |  | Jun-05 |
|  |  |  | Jun-07 |
|  |  |  | Jun-11 |
|  |  |  | Jun-12 (2 counts) |
|  |  |  | Jun-14 (2 counts) |
|  |  |  | Jul-01 |
|  |  |  | Jul-08 |
|  |  |  | Jul-16 |
|  |  |  | Jul-23 |
|  |  |  | Aug-06 |
|  |  |  | Aug-17 |
|  |  |  | Aug-25 |
|  |  |  | Aug-28 |
|  |  |  | Sep-03 |
|  |  |  | Oct-22 |
|  |  |  | Oct-23 |
|  |  |  | Oct-31 |
|  |  |  | Nov-4 (3 counts) |
|  |  |  | Nov-08 |
|  |  |  | Nov-11 |
|  |  |  | Nov-17 (2 counts) |
|  |  |  | Nov-19 (2 counts) |
|  |  |  | Nov-20 |
|  |  |  | Nov-21 (2 counts) |
|  |  |  | Nov-24 |
|  |  |  | Nov-26 |
|  |  |  | Nov-28 |
|  |  |  | Dec-15 |
| 2008 |  |  | Jan-01 |
|  |  |  | Jan-2 (2 counts) |
|  |  |  | Jan-03 |
|  |  |  | Jan-5 (2 counts) |
|  |  |  | Jan-16 |
|  |  |  | Jan-19 (2 counts) |
|  |  |  | Jan-21 |
|  |  |  | Jan-22 |
|  |  |  | Jan 26 (2 counts) |
|  |  |  | Jan-29 |
|  |  |  | Feb-04 |
|  |  |  | Feb-06 |
|  |  |  | Feb-09 |
|  |  |  | Feb-18 |
|  |  |  | Mar-2 (3 counts) |
|  |  |  | Mar-04 |
|  |  |  | Mar-05 |
|  |  |  | Mar-6 (3 counts) |
|  |  |  | Mar-11 |
|  |  |  | Mar-12 |
|  |  |  | Mar-13 |
|  |  |  | Mar-16 (3 counts) |
|  | March 18 - April 6 |  | Mar-19 (8 counts) |
|  |  |  | Mar-20 (2 counts) |
|  |  |  | Mar-21 |
|  |  |  | Mar-22 |
|  |  |  | Mar-24 (4 counts) |
|  |  |  | Mar-26 (3 counts) |
|  |  |  | Mar-27 (3 counts) |
|  |  |  | Mar-28 (6 counts) |
|  |  |  | Mar-29 (2 counts) |
|  |  |  | Mar-30 |
|  |  |  | Mar-31 |
|  |  |  | Apr-02 |
|  |  |  | Apr-9 (2 counts) |
|  |  |  | Apr-12 |
|  |  |  | Apr-14 (2 counts) |
|  |  |  | Apr-15 |
|  |  |  | Apr-16 |
|  |  |  | Apr-18 |
|  |  |  | Apr-27 |
|  |  |  | Apr-28 |
|  |  |  | Apr-29 (3 counts) |
|  |  |  | Apr-30 |
|  |  |  | May-01 |
|  |  |  | May-5 (3 counts) |
|  |  |  | May-9 (2 counts) |
|  |  |  | May-11 |
|  |  |  | May-12 |
|  |  |  | May-14 (2 counts) |
|  |  |  | May-22 |
|  |  |  | May-30 |
|  |  |  | Jun-3 (2 counts) |
|  |  |  | Jun-4 (2 counts) |
|  |  |  | Jun-05 |
|  |  |  | Jun-6 (2 counts) |
|  |  |  | Jun-09 |
|  |  |  | Jun-11 (2 counts) |
|  |  |  | Jun-18 (3 counts) |
|  |  |  | Jun-24 |
|  |  |  | Jun-30 |
|  |  |  | Jul-02 |
|  |  |  | Jul-07 |
|  |  |  | Jul-24 |
|  |  |  | Aug-17 |
|  |  |  | Sep-25 |
|  |  |  | Nov-07 |
|  |  |  | Nov-14 |
|  |  |  | Nov-15 |
|  |  |  | Nov-16 |
|  |  |  | Dec-16 |
|  |  |  | Dec-17 |
|  |  |  | Dec-20 |
|  |  |  | Dec-21 |
|  |  |  | Dec-26 |
|  |  |  | Dec-27 |
|  |  |  | Dec-29 |
|  |  |  | Dec-30 (5 counts) |
|  |  |  | Dec-31 (2 counts) |
| 2009 |  |  | Jan-1 (4 counts) |
|  |  |  | Jan-02 |
|  |  |  | Jan-3 (4 counts) |
|  |  |  | Jan-4 (2 counts) |
|  |  |  | Jan-05 |
|  |  |  | Jan-06 |
|  |  |  | Jan-08 |
|  |  |  | Jan-16 |
|  |  |  | Jan-18 (2 counts) |
|  |  |  | Feb-03 |
|  |  |  | Feb-05 |
|  |  |  | Feb-13 |
|  |  |  | Feb-20 |
|  |  |  | Feb-21 (2 counts) |
|  |  |  | Feb-26 |
|  |  |  | Feb-28 |
|  |  |  | Mar-01 |
|  |  |  | Mar-5 (3 counts) |
|  |  |  | Mar-06 |
|  |  |  | Mar-22 |
|  |  |  | Apr-15 |
|  | May 24 - June 11 |  |  |
|  |  |  | Sep-03 |
|  |  |  | Sep-16 |
|  |  |  | Nov-22 |
| 2010 |  | Jan-14 | Mar-04 |
|  |  |  | Mar-11 |
|  |  |  | Mar-17 |
|  |  |  | Mar-18 (2 counts) |
|  | April 12 - May 3 |  |  |
|  |  |  | May-25 |
|  |  |  | Jun-24 |
|  |  | Aug-02 | Sep-04 |
|  |  |  | Sep-12 |
|  |  |  | Sep-15 |
|  |  |  | Nov-19 (2 counts) |
|  |  |  | Dec-08 |
|  |  |  | Dec-21 |
| 2011 |  |  | Jan-04 |
|  |  |  | Jan-08 |
|  |  |  | Jan-25 |
|  |  |  | Feb-09 |
|  |  |  | Feb-23 (2 counts) |
|  |  |  | Mar-05 |
|  | March 21 - April 7 |  | Mar-22 |
|  |  |  | Mar-23 (2 counts) |
|  |  |  | Apr-04 |
|  |  |  | Apr-07 |
|  |  |  | Apr-08 |
|  |  |  | Apr-09 |
|  |  |  | Apr-10 |
|  |  |  | Apr-11 |
|  |  |  | Apr-12 |
|  |  |  | Apr-18 |
|  |  |  | Jul-12 |
|  |  |  | Aug-01 |
|  |  |  | Aug-07 |
|  |  |  | Aug-18 (6 counts) |
|  |  |  | Aug-19 (3 counts) |
|  |  |  | Aug-20 (5 counts) |
|  |  |  | Aug-24 (5 counts) |
|  |  |  | Aug-25 (2 counts) |
|  |  |  | Aug-28 |
|  |  |  | Sep-07 |
|  |  |  | Sep-27 |
|  |  |  | Sep-29 |
|  |  |  | Oct-03 |
|  |  |  | Oct-29 (2 counts) |
|  |  |  | Oct-30 |

**S3. Analytical Code**

library(strucchange)

library(syuzhet)

library(tseries)

library(forecast)

library(scales)

library(ggplot2)

library(lmtest)

####Assess changes in support over time: ever justified

###create data attitudes

waves<-c(1,2,3,4,5,6,7,8)

ever<-c(94.8, 87.9, 56.4, 49.5, 53.9, 37.6, 45.2, 43.4)

data<-data.frame(Year = waves, DV = ever)

###plot data

ts.ever = ts(data = data$DV, start = c(2004), end=c(2011), frequency=1)

plot(ts.ever, ylab = "ever justified")

####Stationary

nochange_model = lm(ts.ever ~ 1)

AIC(nochange_model)

BIC(nochange_model)

logLik(nochange_model)

mean(abs(nochange_model$residuals))

sqrt(mean(nochange_model$residuals^2))

####Simple linear

linear_model = lm(ts.ever ~ time(ts.ever))

AIC(linear_model)

BIC(linear_model)

logLik(linear_model)

mean(abs(linear_model$residuals))

sqrt(mean(linear_model$residuals^2))

####Fixed breakpoints

bps1 = breakpoints(ts.ever ~ 1, h = 2)

plot(bps1)

bfs1 = breakfactor(bps1, breaks = length(bps1$breakpoints))

breakpoint_model1 = lm(ts.ever ~ bfs1 - 1)

AIC(breakpoint_model1)

BIC(breakpoint_model1)

logLik(breakpoint_model1)

mean(abs(breakpoint_model1$residuals))

sqrt(mean(breakpoint_model1$residuals^2))

#### Model plots

plot(ts.ever, ylab = "Terrorism is ever justified in %")

lines(ts(predict(nochange_model), start = c(2004), end=c(2011), frequency=1)

, col='red'

, lwd=2)

lines(ts(predict(linear_model), start = c(2004), end=c(2011), frequency=1)

, col='darkgreen'

, lwd=2)

lines(ts(predict(breakpoint_model1), start = c(2004), end=c(2011), frequency=1)

, col='orange'

, lwd=2)

legend("topright", legend = c("intercept-only", "linear", "breakpoint", "observed data"), col = c("red", "darkgreen", "orange", "black"), pch = c(19,19,19,19))

####Assess changes in attacks in Jordan

###create data attack

waves<-c(1,2,3,4,5,6,7,8)

attackJ<-c(0,0,3,1,0,0,1,1)

attackJdata<-data.frame(Year = waves, DV = attackJ)

###plot data

ts.attackJ = ts(data = attackJdata$DV, start = c(2004), end=c(2011), frequency=1)

plot(ts.attackJ, ylab = "Attacks in Jordan")

####Stationary

nochange_model = lm(ts.attackJ ~ 1)

AIC(nochange_model)

BIC(nochange_model)

logLik(nochange_model)

mean(abs(nochange_model$residuals))

sqrt(mean(nochange_model$residuals^2))

####Simple linear

linear_model = lm(ts.attackJ ~ time(ts.attackJ))

AIC(linear_model)

BIC(linear_model)

logLik(linear_model)

mean(abs(linear_model$residuals))

sqrt(mean(linear_model$residuals^2))

####Fixed breakpoints

bps1 = breakpoints(ts.attackJ ~ 1, h = 2)

plot(bps1)

bfs1 = breakfactor(bps1, breaks = length(bps1$breakpoints))

breakpoint_model1 = lm(ts.attackJ ~ bfs1 - 1)

AIC(breakpoint_model1)

BIC(breakpoint_model1)

logLik(breakpoint_model1)

mean(abs(breakpoint_model1$residuals))

sqrt(mean(breakpoint_model1$residuals^2))

#### Model plots

plot(ts.attackJ, ylab = "Number of attacks in Jordan")

lines(ts(predict(nochange_model), start = c(2004), end=c(2011), frequency=1)

, col='red'

, lwd=2)

lines(ts(predict(linear_model), start = c(2004), end=c(2011), frequency=1)

, col='darkgreen'

, lwd=2)

lines(ts(predict(breakpoint_model1), start = c(2004), end=c(2011), frequency=1)

, col='orange'

, lwd=2)

legend("topright", legend = c("intercept-only", "linear", "breakpoint", "observed data"), col = c("red", "darkgreen", "orange", "black"), pch = c(19,19,19,19))

###create data injure/fatal

waves<-c(1,2,3,4,5,6,7,8)

injureJ<-c(0,0,163,7,0,0,0,5)

injureJdata<-data.frame(Year = waves, DV = injureJ)

###plot data

ts.injureJ = ts(data = injureJdata$DV, start = c(2004), end=c(2011), frequency=1)

plot(ts.injureJ, ylab = "Casualties in Jordan")

####Stationary

nochange_model = lm(ts.injureJ ~ 1)

AIC(nochange_model)

BIC(nochange_model)

logLik(nochange_model)

mean(abs(nochange_model$residuals))

sqrt(mean(nochange_model$residuals^2))

####Simple linear

linear_model = lm(ts.injureJ ~ time(ts.injureJ))

AIC(linear_model)

BIC(linear_model)

logLik(linear_model)

mean(abs(linear_model$residuals))

sqrt(mean(linear_model$residuals^2))

####Fixed breakpoints

bps1 = breakpoints(ts.injureJ ~ 1, h = 2)

plot(bps1)

bfs1 = breakfactor(bps1, breaks = length(bps1$breakpoints))

breakpoint_model1 = lm(ts.injureJ ~ bfs1 - 1)

AIC(breakpoint_model1)

BIC(breakpoint_model1)

logLik(breakpoint_model1)

mean(abs(breakpoint_model1$residuals))

sqrt(mean(breakpoint_model1$residuals^2))

#### Model plots

plot(ts.injureJ, ylab = "Number of casualties in Jordan")

lines(ts(predict(nochange_model), start = c(2004), end=c(2011), frequency=1)

, col='red'

, lwd=2)

lines(ts(predict(linear_model), start = c(2004), end=c(2011), frequency=1)

, col='darkgreen'

, lwd=2)

lines(ts(predict(breakpoint_model1), start = c(2004), end=c(2011), frequency=1)

, col='orange'

, lwd=2)

legend("topright", legend = c("intercept-only", "linear", "breakpoint", "observed data"), col = c("red", "darkgreen", "orange", "black"), pch = c(19,19,19,19))

####Assess changes in attacks in Israel

###create data attack

waves<-c(1,2,3,4,5,6,7,8)

attackI<-c(37,25,44,54,76,129,8,16)

attackIdata<-data.frame(Year = waves, DV = attackI)

###plot data

ts.attackI = ts(data = attackIdata$DV, start = c(2004), end=c(2011), frequency=1)

plot(ts.attackI, ylab = "Attacks in Israel")

####Stationary

nochange_model = lm(ts.attackI ~ 1)

AIC(nochange_model)

BIC(nochange_model)

logLik(nochange_model)

mean(abs(nochange_model$residuals))

sqrt(mean(nochange_model$residuals^2))

####Simple linear

linear_model = lm(ts.attackI ~ time(ts.attackI))

AIC(linear_model)

BIC(linear_model)

logLik(linear_model)

mean(abs(linear_model$residuals))

sqrt(mean(linear_model$residuals^2))

####Fixed breakpoints

bps1 = breakpoints(ts.attackI ~ 1, h = 2)

plot(bps1)

bfs1 = breakfactor(bps1, breaks = length(bps1$breakpoints))

breakpoint_model1 = lm(ts.attackI ~ bfs1 - 1)

AIC(breakpoint_model1)

BIC(breakpoint_model1)

logLik(breakpoint_model1)

mean(abs(breakpoint_model1$residuals))

sqrt(mean(breakpoint_model1$residuals^2))

#### Model plots

plot(ts.attackI, ylab = "Number of attacks in Israel")

lines(ts(predict(nochange_model), start = c(2004), end=c(2011), frequency=1)

, col='red'

, lwd=2)

lines(ts(predict(linear_model), start = c(2004), end=c(2011), frequency=1)

, col='darkgreen'

, lwd=2)

lines(ts(predict(breakpoint_model1), start = c(2004), end=c(2011), frequency=1)

, col='orange'

, lwd=2)

legend("topright", legend = c("intercept-only", "linear", "breakpoint", "observed data"), col = c("red", "darkgreen", "orange", "black"), pch = c(19,19,19,19))

###create data injure/fatal

waves<-c(1,2,3,4,5,6,7,8)

injureI<-c(726, 227, 236, 185, 61, 261, 19, 14)

injureIdata<-data.frame(Year = waves, DV = injureI)

###plot data

ts.injureI = ts(data = injureIdata$DV, start = c(2004), end=c(2011), frequency=1)

plot(ts.injureI, ylab = "Casualties in Israel")

####Stationary

nochange_model = lm(ts.injureI ~ 1)

AIC(nochange_model)

BIC(nochange_model)

logLik(nochange_model)

mean(abs(nochange_model$residuals))

sqrt(mean(nochange_model$residuals^2))

####Simple linear

linear_model = lm(ts.injureI ~ time(ts.injureI))

AIC(linear_model)

BIC(linear_model)

logLik(linear_model)

mean(abs(linear_model$residuals))

sqrt(mean(linear_model$residuals^2))

####Fixed breakpoints

bps1 = breakpoints(ts.injureI ~ 1, h = 2)

plot(bps1)

bfs1 = breakfactor(bps1, breaks = length(bps1$breakpoints))

breakpoint_model1 = lm(ts.injureI ~ bfs1 - 1)

AIC(breakpoint_model1)

BIC(breakpoint_model1)

logLik(breakpoint_model1)

mean(abs(breakpoint_model1$residuals))

sqrt(mean(breakpoint_model1$residuals^2))

###Plot models

plot(ts.injureI, ylab = "Number of casualties in Israel")

lines(ts(predict(nochange_model), start = c(2004), end=c(2011), frequency=1)

, col='red'

, lwd=2)

lines(ts(predict(linear_model), start = c(2004), end=c(2011), frequency=1)

, col='darkgreen'

, lwd=2)

lines(ts(predict(breakpoint_model1), start = c(2004), end=c(2011), frequency=1)

, col='orange'

, lwd=2)

legend("topright", legend = c("intercept-only", "linear", "breakpoint", "observed data"), col = c("red", "darkgreen", "orange", "black"), pch = c(19,19,19,19))

## CCF of all attacks and support

#test stationary

kpss.test(ts.ever, null = c("Level"))

kpss.test(ts.attackJ, null = c("Level"))

kpss.test(ts.injureJ, null = c("Level"))

kpss.test(ts.attackI, null = c("Level"))

kpss.test(ts.injureI, null = c("Level"))

ccfvalues_attackJ = ccf(ts.attackJ, ts.ever, ylab = "Cross-correlation support for terrorism and number of attacks in Jordan")

ccfvalues_attackJ

ccfvalues_injureJ = ccf(ts.injureJ, ts.ever, ylab = "Cross-correlation support for terrorism and number of casualties in Jordan")

ccfvalues_injureJ

ccfvalues_injureI = ccf(ts.injureI, ts.ever, ylab = "Cross-correlation support for terrorism and number of casualties in Israel")

ccfvalues_injureI

ccfvalues_attackI = ccf(ts.attackI, ts.ever, ylab = "Cross-correlation support for terrorism and number of attacks in Israel")

ccfvalues_attackI

grangertest(ts.injureI~ ts.ever, order=1)

grangertest(ts.ever~ ts.injureI, order=1)

**S4. Differences in Public Support for Terrorism for Sub-samples of Jordanian and Palestinian Respondents**

Below we report mean differences for the variable ‘support for suicide terrorism’ for two sub-samples for the five years where ‘national group’ was recorded in the PGAS: respondents who describe their national group as Jordanian and those who describe their group as Palestinians (M_j_ = mean score Jordanians (SD), M_p_ = mean score for Palestinians (SD)).

2007: t(963) = -1.89, p = .059 (two-sided), M_j_ = 3.72 (1.70), M_p_ = 3.50 (1.75), d = -.12

2008: t(966) = -1.12, p = .262 (two-sided), M_j_ = 3.41 (1.40), M_p_ = 3.31 (1.46), d = -.07

2009: t(961) = -.30, p = .761 (two-sided), M_j_ = 3.75 (1.33), M_p_ = 3.72 (1.40), d = -.02

2010: t(964) = -2.17, p = .030 (two-sided), M_j_ = 3.39 (1.00), M_p_ = 3.23 (1.15), d = -.14

2011: t(965) = .89, p = .373 (two-sided), M_j_ = 3.46 (1.00), M_p_ = 3.52 (1.06), d = .06

The results show a significant difference between national groups in 2010; however, this is a small effect and given the large sample size, the effect size is especially informative. It could be speculated that this difference reflects differential responses to a drop in casualties in Israel between 2009 and 2010. Specifically, comparing Palestinian support for terrorism between 2009 and 2010, expressed support increases (mean difference: -.49, (SE = .077), p < .001; the variable is coded such that lower values indicate stronger support). Having said this, this increase in support between 2009 and 2010 is not reflected in the overall opinion trends. Therefore, we do not believe that the oversampling of Palestinian respondents affects the identified trends and conclusions.

**S5. Targets Jordan**

|  | **Private citizen** | **Military (Foreign)** | **Business** | **Government (Diplomatic)** | **Tourists** |
| --- | --- | --- | --- | --- | --- |
| 2004 |  |  |  |  |  |
| 2005 |  | 1 | 3 |  |  |
| 2006 |  |  |  |  | 1 |
| 2007 |  |  |  |  |  |
| 2008 |  |  |  |  |  |
| 2009 |  |  |  |  |  |
| 2010 | 1 |  |  | 1 |  |
| 2011 |  |  |  |  |  |

**S6. Weapon types Jordan**

|  | **Explosives** | **Firearms** |
| --- | --- | --- |
| 2004 |  |  |
| 2005 | 4 |  |
| 2006 |  | 1 |
| 2007 |  |  |
| 2008 |  |  |
| 2009 |  |  |
| 2010 | 2 |  |
| 2011 |  |  |

**S7. Targets Israel**

|  | **Private citizen** | **Religious Figure** | **Military** | **Business** | **Food or Water Supply** | **Educational Institution** | **Transportation** | **Government** | **Unknown** | **Utilities** | **Government (Diplomatic)** | **Police** | **Journalists Media** | **Maritime** |
| --- | --- | --- | --- | --- | --- | --- | --- | --- | --- | --- | --- | --- | --- | --- |
| 2004 | 6 |  | 5 | 1 |  | 1 | 3 |  |  |  |  |  | 1 | 1 |
| 2005 | 18 |  | 4 | 2 | 1 | 1 | 1 | 1 |  |  |  |  |  |  |
| 2006 | 64 |  | 8 | 3 | 1 | 1 |  |  | 1 | 1 |  |  |  |  |
| 2007 | 38 |  | 6 | 2 |  | 1 |  |  | 1 |  | 1 |  |  |  |
| 2008 | 105 |  | 12 | 4 |  | 2 |  | 1 | 1 | 1 |  | 3 |  |  |
| 2009 | 30 |  | 1 | 1 |  |  |  | 3 |  |  |  | 1 |  |  |
| 2010 | 13 |  |  |  |  | 1 |  |  |  |  |  |  |  |  |
| 2011 | 47 | 2 |  |  |  |  | 2 |  |  |  |  |  |  |  |

**S8. Weapon types Israel**

|  | **Explosives** | **Melee (Knife)** | **Melee (hands, fists)** | **Firearms** | **Vehicle** | **Incendiary** |
| --- | --- | --- | --- | --- | --- | --- |
| 2004 | 18 |  |  |  |  |  |
| 2005 | 23 | 1 |  | 4 |  |  |
| 2006 | 76 | 1 |  | 2 |  |  |
| 2007 | 47 |  | 1 | 1 |  |  |
| 2008 | 123 |  |  | 5 | 1 |  |
| 2009 | 35 |  |  |  | 1 |  |
| 2010 | 14 |  |  |  |  |  |
| 2011 | 47 |  |  | 3 |  | 1 |
